# “Misguided Members of the Profession” and the Presidential Address

**Published:** 1886-07

**Authors:** 


### DANIEL'S Medical Journal,

PUBLISHED MONTHLY AT

AUSTIN, TEXAS.

F. E. DANIEL, M. D., EDITOR AND PUBLISHER

Subscription Price: Two Dollars per Annum in Advance.

COLLABORATORS.

*E. J. Doering, M. D., Chicago.*
*Geo Cupples, M. D.. San Antonio.*
*Odo Betz, M. D., Germany.*
*E. J. Beall, M. D., Fort Worth, Texas.*
*T. C Osborn, M D., Cleburne,* "
*Wm. Penny, M D., New York.*
*H. O. Marcy, M. D., Boston.*
*C. K. Gregg, M. D., Mexico.*
*R. M Swearingen, M. D.. Austin.*
*C H. Wilkinson, M. D., Galveston.*
*J W McLaughlin, M D., Austin.*
*R H. L. Bibb, M. D., Mexico.*

### EDITORIAL.

### "MISGUIDED MEMBERS OF THE PROFESSION" AND THE PRESIDENTIAL ADDRESS.

Custom is a strange thing; and sometimes, however absurd, a custom is quite tenacious.

It is now, and for a long time has been, and perhaps always will be, in this country, the custom to elect to the offices of honor in medical societies not always the most scientific,—not, necessarily, the ablest or the most learned of the members; but such associations, being of a complex character, exhibiting often, more of a social than of a scientific tendency, and with a proneness to lose the equilibrium, and run all to frolic, it happens occasionally that social qualities count first, in making up the estimates for the annual elections; hence the popular man, being the favorite, comes in under the string, several lengths ahead. This is especially apt to be the case, if, to his admirable social qualities be added a fluency of speech or an aptness at off-hand declamation.

Moreover, custom has made the man of choice a representative man, to that extent that the Association, and the profession at large in the State, are judged, and sized up by his utterances,—his mental calibre; and this, not alone on scientific questions. In fact, generally speaking, it is fair to assume that the President of a State Association does represent the profession which thus honors him. But that he always does so, must be looked upon in the light of a *non sequitur.*

Now, in the eighteen years of the existence of the organization known as the Texas State Medical Association, that representative men have, more than once, been elected to the Presidency, no one will deny; and their administration has reflected credit upon the Association; and they have, in their addresses, fairly reflected the sentiments of the body which they represented. But that this has always been the case with reference to the Texas State Medical Association it cannot be shown.

There are, in fact, two notable exceptions; and as one of the gentlemen referred to said, amongst other things, in his presidential address, "it is well that the Association does not hold itself responsible for the opinions or declarations of its individual members,"—we claim, with equal justice, that the association should not always be held responsible for the utterances and opinions of its presiding officer. The same gentleman also said, "many ideas fraught with absurdity find expression in as learned bodies as ours;" and immediately, though unwittingly, gave an emphatic illustration of the truth of his assertion in the following language

"Strange as it may appear, there are those who say they honestly believe, and actually attempt to teach that man is a descendant of, or has ascended from a lower organism. * * * The larger number of our profession hold and teach that man was created in the image of his Maker, full and complete in all the anatomical structure as we are to-day." (Dr. A. P. Brown, *Presidential Address*, *Trans. Tex. S. Med. Soc.* 1884.

We would not disparage the able and distinguished gentlemen whose addresses we quote, for they are prominent, well known, and, as the matter goes, worthy of the distinction conferred upon them by their election to the highest office within the gift of the Association; but we do say that, in our deliberate judgment, the majority of the members of the Association deprecate the introduction of a religious element into the annual addresses. It is thought that such addresses should be of a purely scientific character; and, in these two instances, the gentlemen have disappointed many friends, and surprised all. Not only that, speaking for, and in the name of the Association, they have transcended the bounds of propriety in venturing upon theological grounds, but they have spoken sentiments which do not find response or sympathy in the bosoms of a large majority of the members. Such sentiments, doubtless, are honestly entertained by the distinguished gentlemen, and, doubtless, do honor to their early religious training; but we hold that they are out of place, and untimely, when incorporated into an address before a society whose ends are ostensibly the advancement of medical and cognate sciences.

So reluctant are we to engage in a discussion of this character, that the remarks of President Brown, delivered some two years ago, would have been allowed to pass unnoticed (though we have heard it said by many that the Doctor made a mistake in his estimate of the Association, touching the belief of the members in evolution), were it not that recently there has been an emphatic repetition of this thing—a reiteration of the assertion, in substance, that the members of the Texas State Medical Association reject *in toto* the inspired conceptions of Darwin, Huxley, Herbert Spencer, and others of that school of scientists—the latest revelations of modern science, and that those distinguished men are held in contempt by the profession of Texas; that the great truths enunciated by them, and which are now all but universally accepted, are held in derision by the medical men of a great State. We say, perhaps, we should not have referred to the subject at all, had it not been that at the recent meeting of the State Medical Association at Dallas, our worthy President, Dr. E. P. Becton, emphatically "saw" the theological "*ante*" of Brother Brown, and went him considerably better. Indeed, Dr. Brown, speaking without authority, "went it blind" as to the sentiments of the members; and Dr. Becton certainly "straddled the blind" when he said:

"Now and then some misguided member of the profession accepts the teachings of such men [sic] as Darwin and Huxley; but the great body of American physicians know that these doctrines are as withering, blighting as the winter's frost; that they cramp the genius, freeze the vivid and glowing aspirations of the mind, and clip with unsparing hand, the lofty flights of the intellect"; that they hush the still small voice of conscience here, and damn the soul hereafter." [Here followed a glowing eulogy on the Bible.]

This declaration by the President of the Texas State Medical Association, speaking for, and in behalf of the organized medical profession of Texas, standing unchallenged, thoroughly commits the Association to sentiments which, we believe—nay, know, are foreign to a large majority of the members. It amounts to an emphatic "call;" and hence this article—a showing of hands, as it were.

We have conversed with many of the members, and the result of our observation is, that Dr. Becton stands almost alone in his denunciation of the doctrine of "evolution—natural selection and the survival of the fittest"—solitary, and without support in the disrespectful terms in which he has seen proper to speak of these great—the greatest scientists of this or of any age—men to whom the scientific world owe a heavy debt of gratitude, cheerfully, reverentially acknowledged by them throughout the civilized world! And this journal, aspiring to be the exponent of rational medicine in Texas—reflecting, as it does, to some extent, at least, the sentiments of the State Association, feels called upon to disclaim for itself, and, for a large number of its readers, any participation in, or sympathy with any such opinions! We deny most emphatically that the "great body of the profession" (of Texas, at least) have any such knowledge of the destructive effects of scientific teacnings, to mind, body and soul, as is by Dr. B. ascribed to them. We know, of our own knowledge, many gentlemen who are in perfect sympathy with evolution as opposed to special creation, and who have experienced none of the "blighting," "withering," "damning" and "hushing" influences, so forcibly and so eloquently depicted by our distinguished President. Indeed, they are amongst the most enlightened and progressive of our State Association; and we know, furthermore, that many of them feel sore and hurt at being thus emphatically handed over to Genesis, and represented as despising the men whom the scientific world delights to honor. In the expression of such sentiments, which, as before said, do credit to Dr. Becton's early religious instruction, and which would, doubtless, have won for him a name, great amongst theologians, if thundered from the pulpit, Dr. Becton has transcended fairly the bounds of propriety and good taste, and almost overstepped the limits of forbearance. Such sentiments, we say (and let us not be misunderstood), are out of place in a presidential address before a State Medical Society, however orthodox and appropriate they might be considered elsewhere; and, as the Texas State Medical Association, as such, makes no pretensions to orthodox Christianity, we protest it should not be so completely committed to tenets which give it distinctly that aspect. We are not attacking the orthodoxy of Dr. Becton's belief. He has a perfect right to sustain Genesis from the jump—if he wants to—and to back Adam against protoplasm and the field, so to speak; but let him speak for himself, and not for the State Medical Association. Let him proclaim his religious beliefs from the pulpit, and pour his pathetic pleadings to be held guiltless of "materialism and infidelity" into the ears of sympathizing sisters and brothers; but let him not say that they are "misguided" amongst our fellows who have the capacity to comprehend and the manliness to accept the doctrines of "such men as Darwin and Huxley."

For our part, we see none of that *bugaboo*—"atheism" in the doctrine of man's development from a prime cause;—the admission of such cause, or law of development would seem to demonstrate the existence of an All-Wise Intelligence.

Evolution is no new thing, no "discovery." It is a divine truth, and has existed *ab initio*, eternal and immutable! It is man who has just received its impress upon an awakened consciousness that is new;—he being now, for the first time, sufficiently advanced intellectually,—developed, in accordance with fixed laws of the Creator, to conceive of,—to comprehend the manner and mode of man's progress and development. That Darwin or Huxley, or any other man, should conceive and properly interpret these great truths, amounts to a demonstration of the correctness of their doctrines. Gallilleo, Kepler, Newton, taught no new truths,—hatched up no new theory to account for the systems of worlds and their motion; what they taught had always been so,—but they were the first, so far as we know, to divine the true state of affairs,—to interpret correctly the language of nature, and to expound her governing laws; and we are sure the accepted belief in the laws of gravitation and the relation of the heavenly bodies, to say nothing of our system of geology—militates just as much against Scripture, as does the theory of man's (and the earth's) development against the account of creation given in Genesis.

All this outcry against the doctrine of evolution tending to "atheism" and "infidelity," is on a par with the persecution of Gallilleo; and like it, will pass away with the future,—higher development of the mind of man! The history of civilization furnishes abundant evidence that as man has advanced in intellectual development he has conceived of, and comprehended more and more of the truths and beauties of nature's laws. Alphabets and primers are put into the hands of children. Not until their minds have reached the plane of development which will enable them to comprehend the more abstruse themes,* are they entrusted with books. So with the book of nature. Man is just leaving his childhood (intellectually) behind him, and entering upon a period of existence when doors, shut for all these centuries, yield to the "open sesame" of intellectuality; and when all shall have reached that stage—when what now seems misty, mythical and mixed shall be as clear to the mind as the noon-day's sun, it is to be hoped that man—lay, minister and professional—will be able to entertain a broader conception of Deity than that which debases Him to the stature of a *creature*. Then, and not till then, can man be said to be "an image of his Maker."

*We have often thought that perhaps the Scriptural account of cosmogony was adapted to the then popular comprehension.

